# Altered Brain Functional Network Topology in Lung Cancer Patients After Chemotherapy

**DOI:** 10.3389/fneur.2021.710078

**Published:** 2021-08-02

**Authors:** Jia You, Juan Zhang, Song'an Shang, Wei Gu, Lanyue Hu, Yujie Zhang, Zhenyu Xiong, Yu-Chen Chen, Xindao Yin

**Affiliations:** ^1^Department of Radiology, Nanjing First Hospital, Nanjing Medical University, Nanjing, China; ^2^Department of Neurology, Nanjing Yuhua Hospital, Yuhua Branch of Nanjing First Hospital, Nanjing, China; ^3^Department of Respiratory Medicine, Nanjing First Hospital, Nanjing Medical University, Nanjing, China; ^4^Department of Radiation Oncology, The University of Texas Southwestern Medical Center, Dallas, TX, United States

**Keywords:** lung cancer, chemotherapy, rs-fMRI, graph theory, small-worldness

## Abstract

**Purpose:** This study aimed to explore the topological features of brain functional network in lung cancer patients before and after chemotherapy using graph theory.

**Methods:** Resting-state functional magnetic resonance imaging scans were obtained from 44 post-chemotherapy and 46 non-chemotherapy patients as well as 49 healthy controls (HCs). All groups were age- and gender-matched. Then, the topological features of brain functional network were assessed using graph theory analysis.

**Results:** At the global level, compared with the HCs, both the non-chemotherapy group and the post-chemotherapy group showed significantly increased values in sigma (*p* < 0.05), gamma (*p* < 0.05), and local efficiency, *E*_loc_ (*p* < 0.05). The post-chemotherapy group and the non-chemotherapy group did not differ significantly in the above-mentioned parameters. At the nodal level, when non-chemotherapy or post-chemotherapy patients were compared with the HCs, abnormal nodal centralities were mainly observed in widespread brain regions. However, when the post-chemotherapy group was compared with the non-chemotherapy group, significantly decreased nodal centralities were observed primarily in the prefrontal–subcortical regions.

**Conclusions:** These results indicate that lung cancer and chemotherapy can disrupt the topological features of functional networks, and chemotherapy may cause a pattern of prefrontal–subcortical brain network abnormality. As far as we know, this is the first study to report that altered functional brain networks are related to lung cancer and chemotherapy.

## Introduction

Lung cancer has become a malignant tumor of increasing morbidity, which is a leading cause of cancer-related deaths worldwide (1–3), and consequently brings great social and economic burden to the country (4). There are several major treatment strategies available, including surgery, radiotherapy, and chemotherapy. Chemotherapy is a kind of basic treatment strategy that can prolong the survival of a patient, but there is very little knowledge about functional network alterations as one of research focuses on resting-state functional magnetic resonance imaging (rs-fMRI) accompanied by chemotherapy treatment. Several studies about brain function changes in lung cancer patients have demonstrated the feasibility of fMRI (5, 6). You et al. reported that the dynamic brain activity of patients with chemotherapy significantly decreased in the default-mode network (DMN) region (7), and Hu et al. found that the dynamic functional connectivity variability in patients with chemotherapy was significantly reduced in several brain regions (8). Simo et al. also reported the DMN functional connectivity decreases in patients with lung cancer (9).

Most fMRI studies about lung cancer patients just focus on brain functional connectivity, but few on brain network. In particular, the chemotherapy effects on topological feature damage of the brain functional network associated with lung cancer patients are still elusive. Graph theory analysis can offer a unique framework to measure the topological features of the brain network and has the advantage of studying the brain network on a larger scale than seed-based analysis used in many neuroimaging studies (10). Through these methods, many important topological properties have been displayed, such as small-worldness, which characterizes high global integration and high local specialization between brain regions, and network efficiency, which is characterized by the fault tolerance of the network, as well as degree centrality, which reflects the utilization degree of information resources (11, 12). Recent studies have applied the graph theory analysis to describe the complexity of brain connectivity by quantifying topologies of network representations in some cancer diseases. It was reported that impaired topological features in the brain structural network were revealed in lung cancer patients without chemotherapy and who had no brain metastasis (13). Low-grade glioma patients showed, before and after operation, damaged small-worldness property in the frontal lobe (14). In addition, for breast cancer patients, cancer and chemotherapy can both decrease regional connectivity, leading to a reduced efficiency of brain network (15). In the graph theory model, the brain is represented as a large-scale brain network made up of nodes and edges. The brain regions are defined as nodes, and the anatomical connections or functional correlations between two nodes are defined as edges (16). However, very few studies made an investigation of the topological characteristics of the functional connectomes in patients with lung cancer, and none focus on patients with chemotherapy.

According to prior work and theoretical assumption, this study aimed to assess the abnormalities of small-world properties in patients with lung cancer before or after chemotherapy. We used rs-fMRI to construct the brain functional networks of patients with or without chemotherapy and normal controls and analyze the topological features of their brain networks using graph theory.

## Materials and Methods

### Participants

All subjects provided a written consent before their participation in the study protocol, which was approved by the Medical Research Ethics Committee of Nanjing Medical University.

Forty-four post-chemotherapy and 46 non-chemotherapy lung cancer patients were obtained from the Department of Respiratory Medicine, Nanjing First Hospital, and 49 healthy controls were enrolled through online advertisements (aged between 50 and 70 years, all right-handed) between September 2017 and February 2018. The groups were matched for age and gender. Among the patients with chemotherapy, 32 patients received cisplatin-based therapy and 12 patients received carboplatin-based therapy for at least 6 months (the time from their first chemotherapy to the rs-fMRI scan). All the patients were pathologically diagnosed as lung cancer through surgery, bronchoscope, or percutaneous puncture. The fMRI was performed 1 to 2 weeks after cancer diagnosis. No participants were excluded from the fMRI analysis due to excessive head motion. A summary for the demographic data, histological diagnosis, and tumor stage is provided in Table 1.

**Table 1:** Demographic and clinical characteristics of all subjects.

	**Healthy controls (*n* = 49)**	**Non-chemotherapy (*n* = 46)**	**Post-chemotherapy (*n* = 44)**	***P*-value**
Age, year	60.53 ± 5.05	62.07 ± 9.11	61.77 ± 8.43	0.585^a^
Gender, male/female FD value (mm)	30/19 0.21 ± 0.06	33/13 0.19 ± 0.07	30/14 0.20 ± 0.06	0.540^b^ 0.574^a^
**Histological diagnosis**				
Adenocarcinoma		27	29	
Squamous cell carcinoma		14	11	
Small cell lung cancer		5	4	
**Tumor stage**				
Limited disease		3	1	
Extensive disease		2	3	
I		5	8	
IIA		5	3	
IIB		5	3	
IIIA		3	5	
IIIB		7	4	
IIIC		1	1	
IV		15	16	

*Data are expressed as mean ± SD. P < 0.05 is considered significant*.

*FD, framewise displacement*.

a *The P-value is obtained by using a one-way ANOVA*.

b *The P-value is obtained by using χ^2^ test*.

The participants were excluded from the study if they received prophylactic cranial irradiation, had brain metastatic tumors, or declared a history of known stroke, head injury, Parkinson's disease, Alzheimer's disease, epilepsy, other acute neurological or psychiatric illnesses, major medical illnesses (e.g., anemia, thyroid dysfunction, severe heart diseases, and damaged liver or kidney function), and severe visual or hearing loss.

### MR Acquisition

MRI image data were obtained by a 3.0-Tesla MRI scanner (Ingenia, Philips Medical Systems, Netherlands) with an eight-channel receiver array head coil, and parallel imaging was employed. All scans were acquired with parallel imaging using sensitivity encoding (SENSE) technique, SENSE factor = 2. Parallel imaging acquisition technology can shorten the scanning time and improve the image quality (17). Head motion and scanner noise were alleviated using foam padding and earplugs. The subjects were required to close their eyes, lie down quietly, stay awake, not think about anything special, and avoid any head motion during the scan. Functional images were obtained axially using an echo-planar imaging sequence. The scan range covered the foramen magnum to the top of the skull. The scanning baseline is parallel to the anterior–posterior commissure line (18). The parameters were as follows: repetition time (TR) = 2,000 ms; echo time (TE) = 30 ms; slices = 36; thickness = 4 mm; gap = 0 mm; field of view (FOV) = 240 × 240 mm; matrix = 64 × 64; and flip angle (FA) = 90°. The fMRI sequence was obtained in 8 min and 8 s. Structural images were acquired with a three-dimensional turbo fast echo T1WI sequence with high resolution as follows: TR = 8.1 ms; TE = 3.7 ms; slices = 170; thickness = 1 mm; gap = 0 mm; FA = 8°; matrix = 256 × 256; FOV = 256 × 256 mm. The structural sequence was obtained in 5 min and 29 s.

### Data Pre-processing

Data preprocessing was carried out using Statistical Parameter Mapping 12 (http://www.fil.ion.ucl.ac.uk/spm) and the Graph Theoretical Network Analysis Toolbox for Imaging Connectomics (GRETNA) (2.0.0A http://www.nitrc.org/projects/gretna/) (19). The processing pipeline included the following stages: (1) The first 10 volumes were removed not only for patients to adjust to the environment but also for signal adjustment from the MRI; (2) Slice timing, corrected and realigned, were performed for the remaining 220 images, and head motion >2.0 mm in each direction or rotation angle >2.0° was removed from the analysis; (3) The remaining dataset was normalized to the 3D-T1 data by the diffeomorphic anatomical registration through exponentiated lie algebra methods (reslicing voxel size as 3 × 3 × 3 mm^3^); (4) Detrending and filtering (0.01–0.08 Hz) were performed in turn. Subsequently, several nuisance signals including head motion, the global mean, and signals from white matter and the cerebrospinal fluid were regressed from the data.

Due to the effects of head movements from volume to volume on the brain functional network, framewise displacement (FD) was calculated for every individual to represent the temporal derivative of the movement parameters. There were no significant differences in the FD values among the three groups (Table 1). Moreover, no participant had FD >0.5 mm or more than 35 volumes.

### Functional Connectivity Matrix and Graph Construction

The network was constructed using GRETNA software. First, automated anatomical labeling atlas was adopted to obtain 90 cortical and subcortical regions of interest in the whole brain, and each was taken for a network node. Next, the mean time series was obtained for each region, and the partial correlations of the mean time series between all pairs of the nodes (representing their conditional dependences by excluding the effects of the other 88 regions) were regarded as the edges of the network. This process generated a partial correlation matrix (90 × 90) for each subject, which was converted to a binary matrix according to a predefined threshold. If the absolute partial correlation between regions *i* and area *j* exceeded the threshold, then entry *a*_ij_ = 1; otherwise, *a*_ij_ = 0. The networks of individual subjects were different in the number of edges. To resolve this discrepancy, we applied a range of sparse thresholds S to the correlation matrix to ensure that each graph had the same number of edges. For each participant, *S* was defined as the fraction of the total number of edges remaining in the network; its minimum value was set so that the average node degree of the threshold network was 2log(*N*), where *N* was the number of nodes. The threshold range generated by this process was 0.05 *S* 0.04, and the interval was 0.01. The networks generated by this threshold strategy could estimate the sparse properties of small-worldness and the smallest possible number of false edges. For the brain networks at each sparsity level, we calculated both the global and node network metrics.

### Brain Functional Network Analysis

For the brain function network, the global topological structure of the brain function network and the regional properties of each node were characterized by calculating the global network parameters and the regional node parameters (20, 21). The global parameters examined included small-world parameters, including Cp (clustering coefficient), Lp (characteristic path length), gamma (normalized clustering coefficient), lambda (normalized characteristic path length), and sigma (small-worldness), as well as network efficiency parameters, including *E*_loc_ (local efficiency) and *E*_glob_ (global efficiency). The node parameters examined included BC (betweenness centrality), DC (degree centrality), nodal clustering coefficient, NE (nodal efficiency), nodal local efficiency, and nodal shortest path.

### Statistical Analysis

Statistical comparisons of the demographics were conducted using SPSS 19.0 software package (SPSS, Inc., Chicago, IL, USA). *p* < 0.05 was statistically significant. The area under the curve (AUC) for each network metric was calculated. The AUC calculation range for a general metric was the sparsity range from *S*1 to *Sn*, and the interval was Δ*S*, where S1 = 0.05, S*n* = 0.40, and Δ*S* = 0.01. The AUC provided a summarized scalar for the topological characteristics of the brain network, that is, independent of a single threshold selection, and was sensitive to the topological changes of brain diseases.

The AUCs of all the network metrics of the control group, the non-chemotherapy group, and the post-chemotherapy group were separately statistically analyzed using one-way analysis of variance (ANOVA). If the ANOVA test showed significant differences, we further studied the differences of graph theory parameters of any two groups using two-sample *t*-tests. The nodal characteristics of the three groups are statistically analyzed as detailed above. Correction for multiple comparisons was carried out using the Bonferroni calibration (*P*_Bonferroni_ < 0.05).

## Results

### Alterations in Global Network Organization

**Figure 1:**
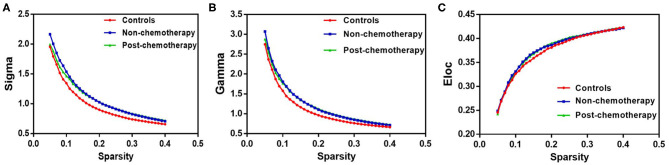
The differences in topological properties of functional networks among the non-chemotherapy and post-chemotherapy groups and the healthy controls. Compared with the controls, the non-chemotherapy group and the post-chemotherapy group showed a significant increase in sigma **(A)**, gamma **(B)**, and *E*_loc_ **(C)**. The post-chemotherapy group and the non-chemotherapy group did not differ significantly in sigma, gamma, and *E*_loc_.

Compared with the healthy controls (HCs), the non-chemotherapy group showed significantly increased values in sigma (*p* = 0.0003), gamma (*p* = 0.0000), and *E*_loc_ (*p* = 0.0002) (Figure 1). The post-chemotherapy group showed significantly increased values in sigma (*p* = 0.0058), gamma (*p* = 0.0028), and *E*_loc_ (*p* = 0.0363) (Figure 1). There were no significantly decreased values in the above-mentioned parameters after comparison. The post-chemotherapy group and the non-chemotherapy group did not differ significantly in sigma, gamma, and *E*_loc_ (Figure 1). The three groups did not differ significantly in Cp, Lp, lambda, and *E*_glob_.

### Alterations in Local Network Metrics – Betweenness Centrality

**Figure 2:**
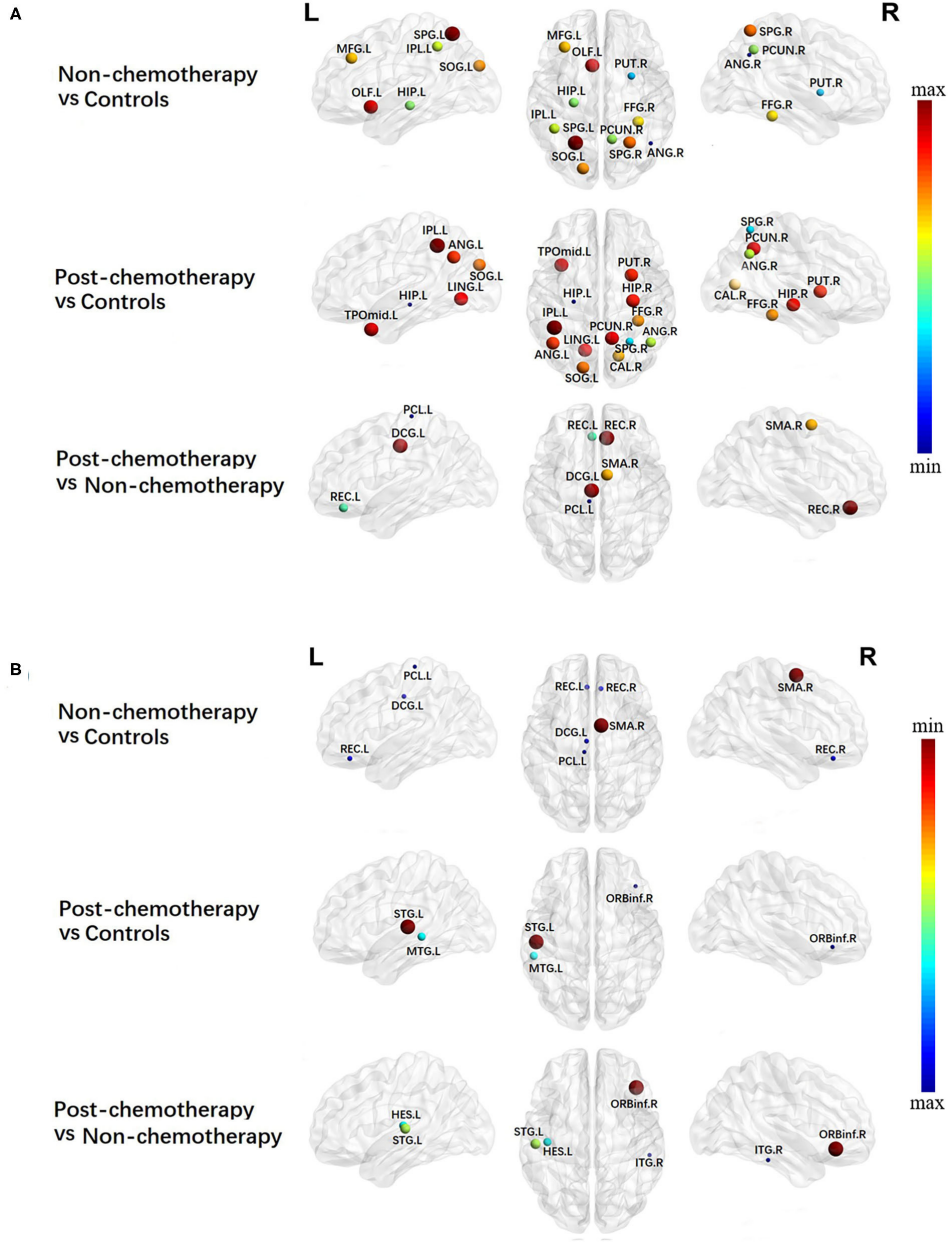
Regions showing increased **(A)** and decreased **(B)** betweenness centrality in non-chemotherapy compared with healthy controls, post-chemotherapy compared with healthy controls, and post-chemotherapy compared with non-chemotherapy. The correction for multiple comparisons was performed using the Bonferroni calibration (*P*_Bonferroni_ < 0.05). The size of the nodes represents *T* value, and the color of the nodes represents *P*_Bonferroni_ value. **(A)** The bluer the color of the node was, the smaller the *P*_Bonferroni_ was, and the redder the color of the node was, the larger the *P*_Bonferroni_ was; **(B)** the redder the color of the node was, the smaller the *P*_Bonferroni_ was, and the bluer the color of the node was, the larger the *P*_Bonferroni_ was_._ The nodes were mapped onto the cortical surfaces using the BrainNet Viewer package (http://www.nitrc.org/projects/bnv).

Compared with the HCs, the non-chemotherapy group showed increased BC in the right angular gyrus (ANG.R), right lenticular nucleus – putamen (PUT.R), left hippocampus (HIP.L), right precuneus (PCUN.R), left inferior parietal but supramarginal and angular gyri (IPL.L), right fusiform gyrus (FFG.R), left middle frontal gyrus, left superior occipital gyrus (SOG.L), right superior parietal gyrus (SPG.R), left olfactory cortex (OLF.L), and left superior parietal gyrus (SPG.L) (Figure 2A; Table 2) as well as decreased BC in the right superior temporal gyrus (SMA.R), left gyrus rectus (REC.L), right gyrus rectus (REC.R), left median cingulate and paracingulate gyri (DCG.L), and left paracentral lobule (PCL.L) (Figure 2B; Table 2).

**Table 2:** Regions showing increased betweenness centrality (BC) and decreased BC in non-chemotherapy compared with controls, post-chemotherapy compared with controls, and post-chemotherapy compared with non-chemotherapy.

**BC increased**			**BC decreased**		
**Brain region**	***T***	***P*_**Bonferroni**_**	**Brain region**	***T***	***P*_**Bonferroni**_**
**Non-chemotherapy*****vs***. **controls**					
Angular_R	3.7675	0.0003^*^	Supp_Motor_Area_R	−4.4787	0.0000^*^
Putamen_R	3.2507	0.0016^*^	Rectus_L	−2.2467	0.0270
Hippocampus_L	2.8787	0.0050^*^	Rectus_R	−2.2242	0.0286
Precuneus_R	2.8491	0.0054^*^	Cingulum_Mid_L	−2.1790	0.0319
Parietal_Inf_L	2.7776	0.0066^*^	Paracentral_Lobule_L	−2.0255	0.0457
Fusiform_R	2.6278	0.0101			
Frontal_Mid_L	2.5984	0.0109			
Occipital_Sup_L	2.5167	0.0136			
Parietal_Sup_R	2.4411	0.0165			
Olfactory_L	2.2054	0.0299			
Parietal_Sup_L	2.0373	0.0445			
**Post-chemotherapy*****vs***. **controls**					
Hippocampus_L	4.0706	0.0001^*^	Temporal_Sup_L	−2.4731	0.0153
Parietal_Sup_R	3.3855	0.0011^*^	Temporal_Mid_L	−2.2955	0.0240
Angular_R	2.9349	0.0042^*^	Frontal_Inf_Orb_R	−2.1910	0.0310
Calcarine_R	2.6239	0.0102			
Fusiform_R	2.5523	0.0124			
Occipital_Sup_L	2.4865	0.0147			
Angular_L	2.3678	0.0200			
Putamen_R	2.3346	0.0218			
Hippocampus_R	2.2841	0.0247			
Lingual_L	2.2503	0.0268			
Precuneus_R	2.2475	0.0270			
Temporal_Pole_Mid_L	2.2138	0.0293			
Parietal_Inf_L	2.0024	0.0482			
**Post-chemotherapy*****vs***. **non-chemotherapy**					
Paracentral_Lobule_L	3.8015	0.0003^*^	Frontal_Inf_Orb_R	−2.7919	0.0064^*^
Rectus_L	3.0269	0.0032^*^	Temporal_Sup_L	−2.4560	0.0160
Supp_Motor_Area_R	2.5925	0.0112	Heschl_L	−2.3260	0.0223
Cingulum_Mid_L	2.1168	0.0371	Temporal_Inf_R	−2.0536	0.0430
Rectus_R	2.0533	0.0430			

*The P_Bonferroni_ values are obtained by using the Bonferroni calibration. P_Bonferroni_ < 0.05 is considered significant*.

* *P_Bonferroni_ < 0.01*.

Compared with the HCs, the post-chemotherapy group showed increased BC in the HIP.L, SPG.R, ANG.R, right calcarine fissure and surrounding cortex, FFG.R, SOG.L, left angular gyrus (ANG.L), PUT.R, right hippocampus (HIP.R), left lingual gyrus, PCUN.R, left temporal pole/middle temporal gyrus (TPOmid.L), and IPL.L (Figure 2A; Table 2) as well as decreased BC in the left superior temporal gyrus (STG.L), left middle temporal gyrus (MTG.L), and right inferior frontal gyrus, orbital part (ORBinf.R) (Figure 2B; Table 2).

Compared with the non-chemotherapy group, the post-chemotherapy group showed increased BC in the PCL.L, REC.L, SMA.R, DCG.L, and REC.R (Figure 2A; Table 2) as well as decreased BC in the ORBinf.R, STG.L, left heschl gyrus, and right inferior temporal gyrus (Figure 2B; Table 2).

### Alterations in Local Network Metrics – Nodal Efficiency

**Figure 3:**
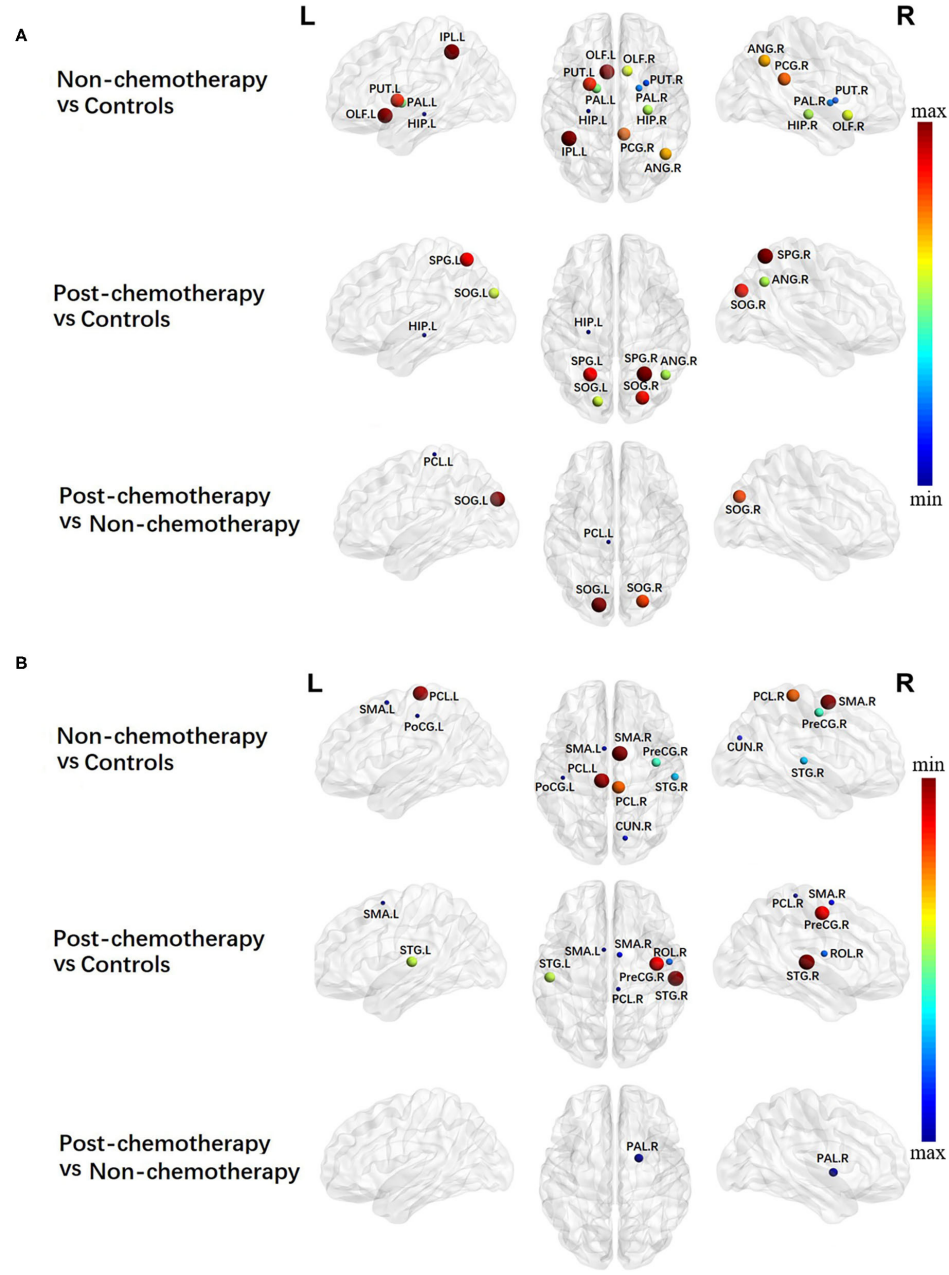
Regions showing increased **(A)** and decreased **(B)** nodal efficiency in non-chemotherapy compared with healthy controls, post-chemotherapy compared with healthy controls, and post-chemotherapy compared with non-chemotherapy. Correction for multiple comparisons was performed using the Bonferroni calibration (*P*_Bonferroni_ < 0.05). The size of the nodes represents the *T* value, and the color of the nodes represents the *P*_Bonferroni_ value. **(A)** The bluer the color of the node was, the smaller the *P*_Bonferroni_ was, and the redder the color of the node was, the larger the *P*_Bonferroni_ was; **(B)** the redder the color of the node was, the smaller the *P*_Bonferroni_ was, and the bluer the color of the node was, the larger the *P*_Bonferroni_ was_._ The nodes were mapped onto the cortical surfaces using the BrainNet Viewer package (http://www.nitrc.org/projects/bnv).

Compared with the HCs, the non-chemotherapy group showed increased NE in the HIP.L, PUT.R, right lenticular nucleus—pallidum (PAL.R), left lenticular nucleus—pallidum, HIP.R, right olfactory cortex (OLF.R), ANG.R, right posterior cingulate gyrus (PCG.R), left lenticular nucleus—putamen (PUT.L), OLF.L, and IPL.L (Figure 3A; Table 3) as well as decreased NE in the SMA.R, PCL.L, right paracentral lobule (PCL.R), right precentral gyrus (PreCG.R), right superior temporal gyrus (STG.R), right cuneus (CUN.R), left supplementary motor area (SMA.L), and left postcentral gyrus (PoCG.L) (Figure 3B; Table 3).

**Table 3:** Regions showing increased nodal efficiency (NE) and decreased NE in non-chemotherapy compared with controls, post-chemotherapy compared with controls, and post-chemotherapy compared with non-chemotherapy.

**NE increased**			**NE decreased**		
**Brain region**	***T***	***P*_**Bonferroni**_**	**Brain region**	***T***	***P*_**Bonferroni**_**
**Non-chemotherapy*****vs***. **controls**					
Hippocampus_L	3.2169	0.0018^*^	Supp_Motor_Area_R	−3.8964	0.0002^*^
Putamen_R	2.9938	0.0035^*^	Paracentral_Lobule_L	−3.8399	0.0002^*^
Pallidum_R	2.9384	0.0042^*^	Paracentral_Lobule_R	−3.5327	0.0006^*^
Pallidum_L	2.6312	0.0100	Precentral_R	−2.9713	0.0038^*^
Hippocampus_R	2.5727	0.0117	Temporal_Sup_R	−2.7554	0.0071^*^
Olfactory_R	2.5020	0.0141	Cuneus_R	−2.4053	0.0181
Angular_R	2.3653	0.0201	Supp_Motor_Area_L	−2.3276	0.0221
Cingulum_Post_R	2.2669	0.0257	Post-central_L	−2.2653	0.0258
Putamen_L	2.1953	0.0306			
Olfactory_L	2.0305	0.0452			
Parietal_Inf_L	2.0074	0.0476			
**Post-chemotherapy*****vs***. **controls**					
Hippocampus_L	3.6235	0.0005^*^	Supp_Motor_Area_L	−4.1959	0.0001^*^
Angular_R	2.7790	0.0066^*^	Paracentral_Lobule_R	−4.0167	0.0001^*^
Occipital_Sup_L	2.7295	0.0076^*^	Supp_Motor_Area_R	−3.4987	0.0007^*^
Occipital_Sup_R	2.2755	0.0252	Rolandic_Oper_R	−2.9300	0.0043^*^
Parietal_Sup_L	2.2524	0.0267	Temporal_Sup_L	−2.7729	0.0067^*^
Parietal_Sup_R	2.0641	0.0419	Precentral_R	−2.6245	0.0102
			Temporal_Sup_R	−2.6080	0.0106
**Post-chemotherapy*****vs***. **non-chemotherapy**					
Paracentral_Lobule_L	3.6192	0.0005^*^	Pallidum_R	−2.7762	0.0067^*^
Occipital_Sup_R	2.5205	0.0135			
Occipital_Sup_L	2.2700	0.0256			

*The P_Bonferroni_ values are obtained by using the Bonferroni calibration. P_Bonferroni_ < 0.05 is considered significant*.

* *P_Bonferroni_ < 0.01*.

Compared with the HCs, the post-chemotherapy group showed increased NE in the HIP.L, ANG.R, SOG.L, right superior occipital gyrus (SOG.R), SPG.L, and SPG.R (Figure 3A; Table 3) as well as decreased NE in the right supplementary motor area (STG.R), PCL.R, SMA.L, right rolandic operculum (ROL.R), SMA.R, PreCG.R, and STG.L (Figure 3B; Table 3).

Compared with the non-chemotherapy group, the post-chemotherapy group showed increased NE in the PCL.L, SOG.R, and SOG.L (Figure 3A; Table 3) as well as decreased NE in the PAL.R (Figure 3B; Table 3).

### Alterations in Local Network Metrics – Degree Centrality

**Figure 4:**
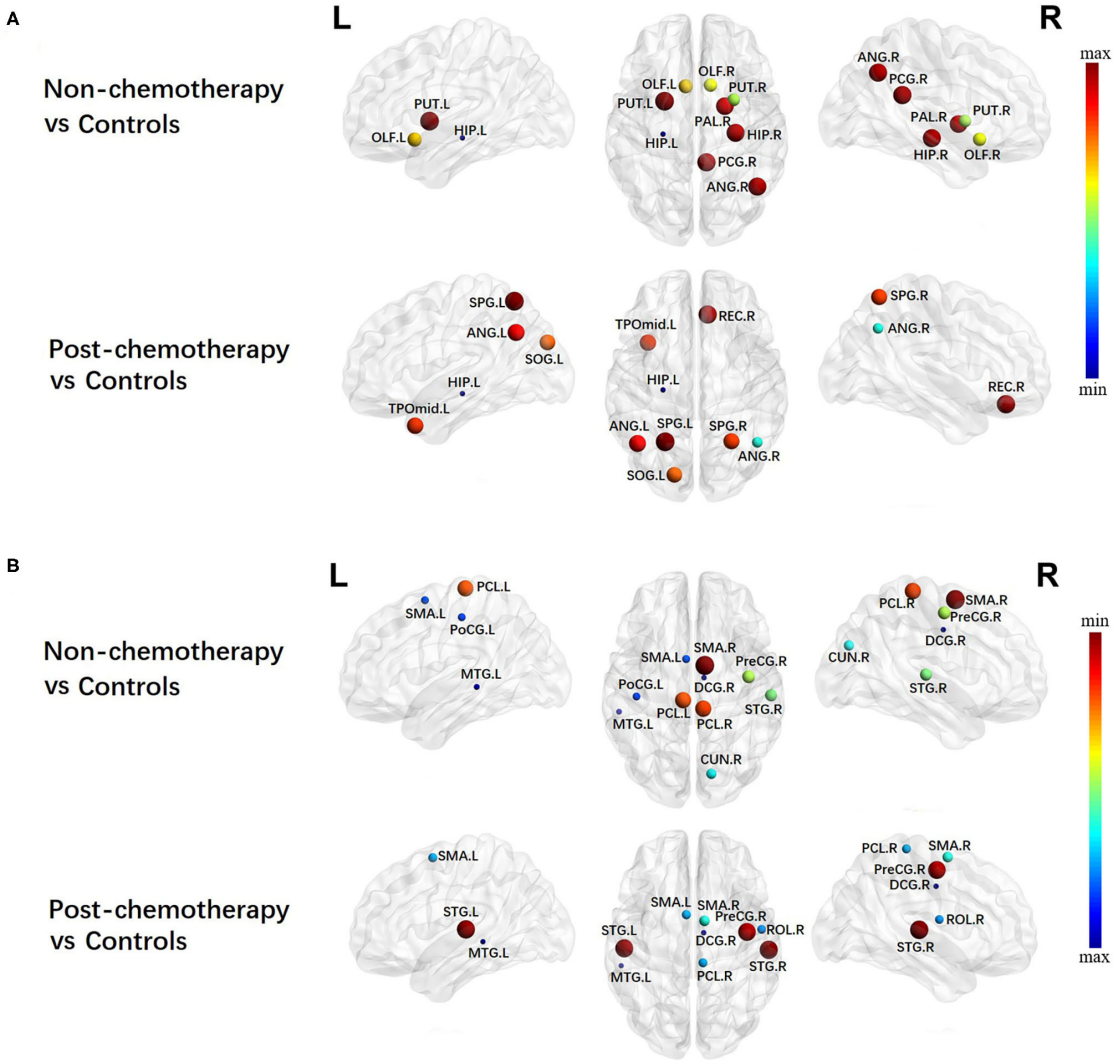
Regions showing increased **(A)** and decreased **(B)** degree centrality in non-chemotherapy compared with healthy controls and post-chemotherapy compared with healthy controls. Correction for multiple comparisons was performed using the Bonferroni calibration (*P*_Bonferroni_ < 0.05). The size of the nodes represents the *T* value, and the color of the nodes represents the *P*_Bonferroni_ value. **(A)** The bluer the color of the node was, the smaller the *P*_Bonferroni_ was, and the redder the color of the node was, the larger the *P*_Bonferroni_ was. **(B)** The redder the color of the node was, the smaller the *P*_Bonferroni_ was, and the bluer the color of the node was, the larger the *P*_Bonferroni_ was_._ The nodes were mapped onto the cortical surfaces using the BrainNet Viewer package (http://www.nitrc.org/projects/bnv).

Compared with the HCs, the non-chemotherapy group showed increased DC in the HIP.L, PUT.R, OLF.R, OLF.L, PAL.R, HIP.R, ANG.R, PCG.R, and PUT.L (Figure 4A; Table 4) as well as decreased DC in the SMA.R, PCL.R, PCL.L, PreCG.R, STG.R, CUN.R, SMA.L, PoCG.L, MTG.L, and right median cingulate and paracingulate gyri (DCG.R) (Figure 4B; Table 4).

**Table 4:** Regions showing increased degree centrality(DC) and decreased DC in non-chemotherapy compared with controls and post-chemotherapy compared with controls.

**DC increased**			**DC decreased**		
**Brain region**	***T***	***P*_**Bonferroni**_**	**Brain region**	***T***	***P*_**Bonferroni**_**
**Non-chemotherapy*****vs***. **controls**					
Hippocampus_L	3.9816	0.0001^*^	Supp_Motor_Area_R	−4.0454	0.0001^*^
Putamen_R	2.9023	0.0046^*^	Paracentral_Lobule_R	−3.6670	0.0004^*^
Olfactory_R	2.7860	0.0065^*^	Paracentral_Lobule_L	−3.6458	0.0004^*^
Olfactory_L	2.6634	0.0091^*^	Precentral_R	−3.1407	0.0023^*^
Pallidum_R	2.1524	0.0340	Temporal_Sup_R	−3.0495	0.0030^*^
Hippocampus_R	2.0935	0.0390	Cuneus_R	−2.7922	0.0064^*^
Angular_R	2.0793	0.0403	Supp_Motor_Area_L	−2.4542	0.0160
Cingulum_Post_R	2.0602	0.0422	Post-central_L	−2.4445	0.0164
Putamen_L	1.9901	0.0495	Temporal_Mid_L	−2.1561	0.0337
			Cingulum_Mid_R	−2.0877	0.0396
**Post-chemotherapy*****vs***. **controls**					
Hippocampus_L	4.5913	0.0000^*^	Temporal_Sup_R	−3.8341	0.0002^*^
Angular_R	3.5949	0.0005^*^	Temporal_Sup_L	−3.8072	0.0003^*^
Occipital_Sup_L	2.6131	0.0105	Precentral_R	−3.7487	0.0003^*^
Parietal_Sup_R	2.5434	0.0127	Supp_Motor_Area_R	−2.7253	0.0077^*^
Temporal_Pole_Mid_L	2.4839	0.0148	Supp_Motor_Area_L	−2.5306	0.0131
Angular_L	2.3761	0.0196	Paracentral_Lobule_R	−2.5164	0.0136
Rectus_R	2.1235	0.0364	Rolandic_Oper_R	−2.4736	0.0152
Parietal_Sup_L	2.0448	0.0438	Cingulum_Mid_R	−2.0100	0.0474
			Temporal_Mid_L	−1.9914	0.0494

*The P_Bonferroni_ values are obtained by using the Bonferroni calibration. P_Bonferroni_ < 0.05 is considered significant*.

* *P_Bonferroni_ < 0.01*.

Compared with the HCs, the post-chemotherapy group showed increased DC in the HIP.L, ANG.R, SOG.L, SPG.R, TPOmid.L, ANG.L, REC.R, and SPG.L (Figure 4A; Table 4) as well as decreased DC in the STG.R, STG.L, PreCG.R, SMA.R, SMA.L, PCL.R, ROL.R, DCG.R, and MTG.L (Figure 4B; Table 4). The post-chemotherapy group and the non-chemotherapy group did not differ significantly in DC.

The three groups did not differ significantly in nodal clustering coefficient, nodal local efficiency, and nodal shortest path.

## Discussion

In this study, our main findings were summarized as follows: (1) at the global level, compared with the HCs, the functional connections of non-chemotherapy patients had significant differences in topological properties, suggesting that cancer may promote the development of central nervous system toxicity; and (2) at the nodal level, when non-chemotherapy patients were compared with the HCs, abnormal nodal centralities were mainly found in widespread brain regions. These results provided clear evidence for the destruction of the whole-brain functional network topology in lung cancer patients without chemotherapy and helped expand the understanding of the reorganization of the brain injury network after cancer. Besides this, compared with the non-chemotherapy group, the post-chemotherapy group primarily observed significantly decreased nodal centralities in the prefrontal–subcortical regions. The result provided explicit evidence of the disrupted topology of brain functional networks in lung cancer patients after chemotherapy.

### Alterations in Global Network Organization

Our results demonstrated that the brain functional networks of patients before and after chemotherapy as well as of the HCs showed a prominent small-world property. Networks with small-world properties ensure higher information-processing efficiency for both locally specialized and globally integrated processing (22). Despite the common small-world properties, the sigma, gamma, and *E*_loc_ were significantly higher in lung cancer patients with or without chemotherapy than the HCs; however, the post-chemotherapy and the non-chemotherapy patients did not differ significantly in the sigma, gamma, and *E*_loc_. The small-worldness measured by sigma essentially reflected the balance of differentiation and integration in the network (23). The current results may reflect the imbalance in the differentiation and integration of brain networks in lung cancer patients. Therefore, it was further confirmed that cancer could interfere with the neural network structure, which also showed that sigma could be used as a quantitative and physiological indicator to assist in clinical diagnosis. Altered gamma suggested that the network connectivity and the degree of the network colony were disturbed, indicating that the highly local integrity and the integrity of brain connections in lung cancer patients can be impaired. *E*_loc_ is the measure of local network connectivity, so the increase of *E*_loc_ in lung cancer patients may represent disrupted information processing among distant brain areas (24). We also identified higher local efficiency, indicative of compensatory mechanisms that form clusters to preserve efficient communication (25). These results provided unequivocal evidence of a topological alteration of the functional connectome in lung cancer patients. These neuroimaging findings suggested that cancer might lead to the development of toxicity in the brain.

### Alterations in Local Network Metrics

Besides the global topology alteration in lung cancer patients with or without chemotherapy, we also found abnormal nodal centrality in these patients. Nodes with high betweenness/degree in structural networks suggest a high interaction between regions and the potential to participate in a much functional interaction (26). High betweenness/degree nodes was reported in temporal regions in acute lymphoblastic leukemia (ALL) patients. The results suggested that the brain network of ALL patients has fewer highly interactive nodes in the temporal areas (27). In the present study, we mainly found altered betweenness/degree nodes in the entire brain regions in lung cancer patients, indicating that cancer may cause a widespread interaction change of brain network in lung cancer patients. Besides this, we mainly found altered nodal efficiency nodes in widespread brain regions in lung cancer patients. Nodal efficiency can quantify the importance of nodes for communication in the brain network (28). Therefore, according to our nodal efficiency results, we consider that cancer may cause partly brain network disruptions in lung cancer patients. Liu et al. reported that cancer can cause central neurotoxicity through studying the topology of structural network in lung cancer patients (29), which are consistent with our current results.

Generally, patterns of nodal alterations were consistent using betweenness, degree, and nodal efficiency. These characteristics can be used to reflect the roles of nodes in information transport and integration across the network. If these measures indicate abnormalities, we should consider that brain network disruption can occur (30). Our results showed that abnormal nodal centralities were found in the entire brain regions in lung cancer patients with chemotherapy. Chemotherapy may also cause widespread brain network disruptions in lung cancer patients. Simo et al. revealed that lung cancer patients with chemotherapy showed decreased functional connectivity in the DMN (9). Besides this, previous fMRI studies also reported that broken regional network features were shown in the frontal, temporal, and striatal regions in cancer patients with chemotherapy (31, 32). These findings accorded with ours, thus providing further evidence for the neuropathological mechanism associated with chemotherapy.

According to our results, we speculate that chemotherapy can cause brain network destruction of the prefrontal and subcortical regions. These results provide the first evidence of brain network changes in the prefrontal and subcortical regions in lung cancer patients with chemotherapy. Interestingly, we found that increased nodal characteristics occurred in the frontal and parietal regions. This result might be attributed to compensatory efforts: if the brain activity of one region decreases, the brain activity of another region will increase. When nerves are damaged, compensation mechanisms might be activated first, and then a new neural network will gradually form to produce functional replacement (33). However, further work is needed to prove this hypothesis.

There were several limitations in the current study. First, this research was a preliminary study, and the sample size was relatively small. In addition, the confounding effects of different pathologies, disease stage, chemotherapy regimen, and disease monitoring will limit the study findings. In future studies, a larger sample size should be used to verify these results and to explore more clinical significance. Second, although we discovered that chemotherapy would cause brain network changes in the prefrontal–subcortical regions, we did not have neuropsychological assessment information for all patients, so we cannot perform any relevant analysis. It is reported that the prefrontal–subcortical systems play important roles not only in cognitive control but also in emotion regulation (34, 35). Thus, we will make further exploration in the cognition–emotion direction in the future. Third, we divided the whole brain into 90 regions to conduct the functional brain networks. Further studies are required to find the most appropriate brain parcellation strategy or spatial scale for the characterization of network topology as different parcellation schemes or as different spatial scales exhibit distinct topological architectures. Furthermore, Hu et al. revealed that the functional connectivity within the executive control network was reduced in lung cancer patients after chemotherapy with dynamic connectivity analysis (8). So far, there have been a few studies on lung cancer patients before or after chemotherapy using brain dynamic network. Thus, we will further study our data from the perspective of dynamics and find out the neurophysiological mechanisms that affect brain function after lung cancer and chemotherapy.

## Conclusions

In summary, this study demonstrates that lung cancer and chemotherapy can cause alterations in specific brain network topological properties. Especially the prefrontal–subcortical regions of lung cancer patients show obvious brain network topological property alterations after chemotherapy. Those changes in the specific topological metrics may provide a novel insight regarding the neurobiological mechanisms in these patients and highlight critical areas for future research related to the prefrontal–subcortical regions to the neurologic effects in lung cancer patients with chemotherapy.

## Data Availability Statement

The original contributions presented in the study are included in the article/supplementary material, further inquiries can be directed to the corresponding authors.

## Ethics Statement

The studies involving human participants were reviewed and approved by Medical Research Ethics Committee of Nanjing Medical University. The patients/participants provided their written informed consent to participate in this study.

## Author Contributions

JY and JZ designed the experiment, analyzed the data, and drafted the paper for the work. SS, WG, LH, and YZ helped to acquire the clinical and fMRI data. ZX helped to revise the paper critically for important intellectual content. XY and Y-CC did the financial support, review, and final approval of the paper to be published. All authors have read and approved the final manuscript.

## Conflict of Interest

The authors declare that the research was conducted in the absence of any commercial or financial relationships that could be construed as a potential conflict of interest.

## Publisher's Note

All claims expressed in this article are solely those of the authors and do not necessarily represent those of their affiliated organizations, or those of the publisher, the editors and the reviewers. Any product that may be evaluated in this article, or claim that may be made by its manufacturer, is not guaranteed or endorsed by the publisher.
